# Complete Genome Sequences of Enterovirus D68 Clade A and D Strains in the Philippines

**DOI:** 10.1128/MRA.00709-21

**Published:** 2021-09-30

**Authors:** Michiko Okamoto, Masahiro Sakamoto, Clyde Dapat, Mayuko Saito, Mariko Saito-Obata, Raita Tamaki, Socorro P. Lupisan, Beatriz P. Quiambao, Hitoshi Oshitani

**Affiliations:** a Department of Virology, Tohoku University Graduate School of Medicine, Sendai, Japan; b Research Institute for Tropical Medicine (RITM), Muntinlupa, Philippines; Portland State University

## Abstract

Complete genome sequences were determined for 4 clade A and 12 clade D enterovirus D68 strains detected in nasopharyngeal swabs from children with acute respiratory illness in the Philippines. These sequence data will be useful for future epidemiological monitoring, including watching for viral evolution.

## ANNOUNCEMENT

Enterovirus D68 (EV-D68) belongs to the family *Picornaviridae* and is mainly associated with acute respiratory infections, including severe lower respiratory illnesses (1, 2). EV-D68 has been divided into four clades, A, B, C, and D, based on phylogenetic analysis of the VP1 capsid protein sequences. Previous reports have shown that clade D viruses diverged from clade A after 2014 (3–5). In the Philippines, clade D viruses have been detected periodically since 2008, along with clade A and B viruses (6–8). In 2018, 73.7% of cases (14/19) belonged to clade D, while clade A viruses have not been detected since 2016 (9). The analysis of EV-D68 in our previous studies was performed using partial genome sequencing (6–9). Here, we describe the complete genome sequences of 4 clade A viruses and 12 clade D viruses. We obtained informed consent from the guardians of all participants. This study was approved by the institutional review board of the Research Institute for Tropical Medicine (RITM) and the Ethics Committee of Tohoku University Graduate School of Medicine.

We selected EV-D68-positive nasopharyngeal samples (clades A and D) stored at −80°C from our previous studies (6–9). Viral RNA was extracted using the QIAamp viral RNA minikit (Qiagen). cDNA was generated using SuperScript III reverse transcriptase (Thermo Fisher Scientific) and primers specific to the 3′ terminal region, EV-D68_7333AR and D68_7333BR (10). Two overlapping PCR products encompassing the entire genome were generated using the SequalPrep long polymerase kit (Thermo Fisher Scientific) and EV-D68-specific primers (10). Libraries were constructed using the TruSeq Nano DNA high-throughput library prep kit (Illumina). Paired-end (2 × 151-bp) sequencing was performed on a NovaSeq platform. The raw data were processed using the CLC Genomics Workbench v20.0.4. The sequence reads were sorted by barcode and trimmed using the Trim Reads tool (quality limit = 0.01 and ambiguous trim = 0). The reads were mapped to the reference genome 2012-12225 (GenBank accession number KT285319; clade D) or USA/WI/2009-23248 (MN240519; clade A) using the Map Reads to Reference tool (11). A range of 27,067,922 to 41,049,786 paired-end reads was obtained for each sample (Table 1). The genome lengths of clade A and clade D viruses ranged from 7,323 to 7,341 nucleotides and 7,331 to 7,347 nucleotides, respectively (Table 1). The sequences were aligned using the ClustalW program in MEGA v7.0.26 software (12).

**TABLE 1:** Characteristics of the complete genome sequences of enterovirus D68

Strain name	Collection yr	Clade	Length (nt)^*a*^	Mean coverage (×)	GC content (%)	No. of reads	GenBank accession no.	Sequence Read Archive accession no.
TTa-08-Ph561	2008	D	7,345	721,607	43.6	39,221,310	LC629436	DRR290888
TTa-11-Ph224	2011	A	7,341	483,009	45.3	27,067,922	KX789259.2	DRR290889
TTa-11-Ph272	2011	A	7,326	539,946	44.8	29,216,542	LC629437	DRR290890
TOp-12-Ph146	2012	D	7,347	716,609	43.7	41,049,786	LC629438	DRR290891
TEv-13-Ph137	2013	D	7,345	754,494	43.0	40,919,452	LC629439	DRR290892
TEv-13-Ph173	2013	D	7,331	556,697	44.0	31,184,658	LC629440	DRR290893
TBp-13-Ph209	2013	D	7,345	547,823	43.3	29,362,644	LC629441	DRR290894
TB9-15-Ph380	2015	A	7,323	562,271	43.6	31,531,112	LC629442	DRR290895
TB6-15-Ph427	2015	A	7,326	518,999	43.6	28,072,258	LC629443	DRR290896
TB8-15-Ph508	2015	A	7,338	550,706	43.8	29,741,840	LC629444	DRR290897
TB5-16-Ph232	2016	D	7,333	673,218	44.4	37,958,344	LC629445	DRR290898
TB5-16-Ph262	2016	D	7,332	498,668	44.6	27,844,200	LC629446	DRR290899
TB5-17-Ph282	2017	D	7,333	701,096	44.5	39,897,452	LC629447	DRR290900
TB5-18-Ph204	2018	D	7,345	646,862	45.5	41,004,934	LC629448	DRR290901
TB5-18-Ph483	2018	D	7,345	679,355	43.6	36,960,002	LC629449	DRR290902
TB5-18-Ph631	2018	D	7,345	515,932	44.0	27,821,086	LC629450	DRR290903

a nt, nucleotides. Differences in length among clades occurred only in the variable region of the 5′ untranslated region.

**FIG 1:**
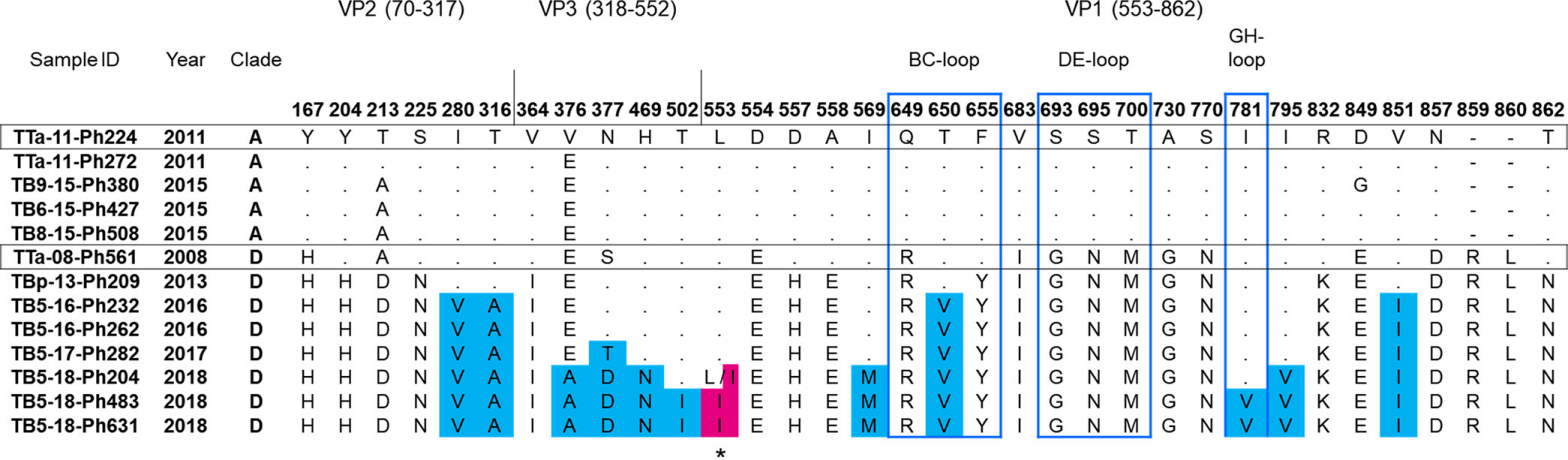
Amino acid substitution of the structural protein of EV-D68. The P1 region sequences (except VP4) of the EV-D68 strains of clades A and D analyzed in this study were aligned, and the numbering was based on strain Fermon (GenBank accession number AY426531.1). Representative strains of clades A and D are shown in open black boxes. The positions where sequences had amino acid residues identical to TTa-11-Ph224 (clade A) are indicated by dots. Solid blue and magenta indicate nonsynonymous substitutions in clade D in a community in Biliran Island after 2016. The open blue boxes represent the BC, DE, and GH loop regions. The asterisk indicates the protease cleavage site for VP3/VP1.

Clade D viruses collected after 2016 contained nonsynonymous substitutions in 17 locations, 12 of which were distributed in VP1 to VP3 (Fig. 1). Of these amino acid substitutions, two were located in the BC and GH loops of the antigenic sites of VP1. An L553I substitution located in the VP3/VP1 cleavage site was observed in clade D viruses detected in 2018. In sample TB5-18-Ph204, the ratio of the amino acid leucine to isoleucine was 7:3, based on a single nucleotide polymorphism at position 2368, with 70% of the reads thymine and 30% adenine. Isoleucine at the VP3/VP1 cleavage site was only found in one 2014 German EV-D68 strain that was deposited in GenBank (accession number KP745741.2). Global surveys of complete genomes are needed to understand the clade shift and evolution of EV-D68.

### Data availability.

The sequences were deposited in GenBank under the accession numbers LC629436 to LC629450, and the raw reads can be found in the NCBI Sequence Read Archive under the accession numbers PRJDB11586 (BioProject), SAMD00319097 to SAMD00319112 (BioSample), and DRR290888 to DRR290903 (SRA).
